# Application of Laser Systems for Detection and Ranging in the Modern Road Transportation and Maritime Sector

**DOI:** 10.3390/s22165946

**Published:** 2022-08-09

**Authors:** Nikola Lopac, Irena Jurdana, Adrian Brnelić, Tomislav Krljan

**Affiliations:** 1 Faculty of Maritime Studies, University of Rijeka, 51000 Rijeka, Croatia; 2 Center for Artificial Intelligence and Cybersecurity, University of Rijeka, 51000 Rijeka, Croatia

**Keywords:** lidar, laser sensors, remote sensing, transportation, road transportation, autonomous vehicles, maritime sector, data analysis, deep learning, object detection

## Abstract

The development of light detection and ranging (lidar) technology began in the 1960s, following the invention of the laser, which represents the central component of this system, integrating laser scanning with an inertial measurement unit (IMU) and Global Positioning System (GPS). Lidar technology is spreading to many different areas of application, from those in autonomous vehicles for road detection and object recognition, to those in the maritime sector, including object detection for autonomous navigation, monitoring ocean ecosystems, mapping coastal areas, and other diverse applications. This paper presents lidar system technology and reviews its application in the modern road transportation and maritime sector. Some of the better-known lidar systems for practical applications, on which current commercial models are based, are presented, and their advantages and disadvantages are described and analyzed. Moreover, current challenges and future trends of application are discussed. This paper also provides a systematic review of recent scientific research on the application of lidar system technology and the corresponding computational algorithms for data analysis, mainly focusing on deep learning algorithms, in the modern road transportation and maritime sector, based on an extensive analysis of the available scientific literature.

## 1. Introduction

Different sensors and measuring instruments contribute significantly to today’s technologies. Data collection represents the basis of building, developing, and implementing various systems. Due to its efficiency, speed, and flexibility in the modern transportation sector, the application of the light detection and ranging (lidar) system stands out as one of the leading solutions for measuring various parameters in a wide range of activities. Object detection, a significant factor in modern transportation, especially in autonomous vehicles, is more detailed and precise when using the lidar system to supplement existing radio detection and ranging (radar), ultrasound, and other sensors. The system is characterized by unique characteristics such as independence from external light for detection purposes, high resolution of detected points, and speed of operation. The research in this field, reviewed and discussed in this paper, gives great promise for the further development and application of lidar technology in the modern transportation sector.

This paper provides a comprehensive overview of various lidar system applications in modern road transportation and the maritime sector. For this purpose, we conducted an extensive literature review by first performing a search in the Web of Science Core Collection database for papers on these topics, published in the last 10 years. The search was conducted using the “lidar” keyword combined with other keywords, including “road transportation”, “autonomous driving”, “maritime”, and “autonomous ship”. The selection of the reduced set of found papers was made by taking into account paper relevance, the number of citations, publication source indicators, and recent year of publication. Moreover, the selection procedure also included choosing those papers addressing specific areas of lidar application within the main topics of road transportation and the maritime sector, in order to achieve adequate representation of particular areas as much as possible, thus providing a complete overview of the field. The selected papers included several important review papers in this field, while most were specific studies, focusing on those recently published in relevant scientific journals, in order to better present the current state-of-the-art.

Most review papers in the field of lidar system application in road transportation focus on a specific, narrow application area. On the other hand, this paper provides a comprehensive overview of applications, analyzing different areas within the main topic. Moreover, we also provide a research background by briefly describing the lidar system technology, operating principles, different system types, measurement principles, and system parameters. Furthermore, we also extend the analysis from the lidar application in modern road transportation to the maritime sector, logically connecting these two fields and emphasizing their specifics, while highlighting common features and challenges, which has not been adequately addressed in previous review papers on these topics, to the best of the authors’ knowledge. Finally, we also critically assess the current challenges and limitations of the technology and discuss future trends and developments.

This paper is organized into the following main sections:

1. Introduction
2. Lidar System
3. Application of Lidar System in Road Transportation
4. Application of the Lidar System in the Maritime Sector
5. Challenges and Future Trends
6. Conclusions

Section 2 describes the lidar system as a core laser system for detecting objects. The main types, architectures, and important parameters are briefly described. Next, Section 3 goes into depth about the topic, explaining the application of the lidar system in autonomous driving, the detection of obstacles, the impact of weather conditions on the system itself, and the detection of roads and objects on and along the road. Furthermore, Section 4 addresses the existing applications of the lidar system in the maritime sector, from object detection to other diverse applications. Finally, Section 5 presents and discusses current challenges and future trends in applying the lidar system in modern transportation, while Section 6 summarizes the conclusions.

## 2. Lidar System

The most generic form of the system called lidar (abbreviated for light detection and ranging, or laser imaging, detection, and ranging) is described here to provide an understanding of the working principle of a laser system for detecting objects and their distances. Lidar works by scanning its field of view (FoV) with one or several laser beams and represents a method for determining the distance of an object from the laser signal transmitter. The scanning system steers laser beams at different horizontal and vertical angles. The transmitted light, i.e., the optical signal from the laser whose waveform intensity, phase, or frequency have been modulated, is reflected from a surface, object, or particle. The signal then returns to the receiver, where the distance is calculated based on the time of flight (ToF), i.e., the time required for the reflected signal to return to the receiver. As it is an optical signal, the laws of geometric optics apply, so, depending on the transmission medium, the optical signal’s propagation speed is close to the speed of light.

**Figure 1:**
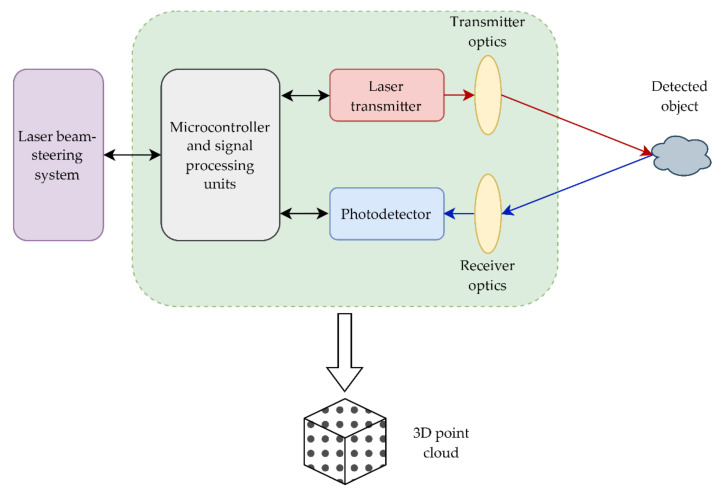
Conceptual representation of the lidar system operating principle.

Furthermore, the wavelength is usually changed during transmission for the device to recognize the transmitted or received signal. The receiver optical lens acts like a telescope and focuses the reflected optical signal onto the photodetector that converts the collected photons returning from the environment into the electronic signal, using the photoelectric effect [1,2]. After that, the signal is filtered according to the variable output/input characteristics and processed for further analysis or storage [3]. Signal processing techniques are used to account for differences in reflected energy caused by surface materials and the environment between the transmitter and receiver [1]. Finally, the lidar outputs contain three-dimensional (3D) point clouds, corresponding to the scanned environment and intensities corresponding to the reflected laser energies [1]. A diagram representation in Figure 1 depicts the described basic concept of the lidar operating principle.

The light sources once used in early experiments on lidar systems were mercury and sodium lamps [3], which were replaced entirely by modern lasers, i.e., light amplification by stimulated emission of radiation. The emitted light can also be outside the visible spectrum, including infrared, ultraviolet, and X-rays [3], but for lidar purposes, the most commonly used is 850–1550 nm range in the infrared spectrum [1]. The range can be divided into two sub-ranges: laser diodes are used at 800–950 nm, while optical-based lasers from the telecommunications industry are used at 1000–1550 nm [4]. The laser is selected depending on the purpose and the location of its use, according to various parameters such as peak power, power consumption, operating temperature, bandwidth, wavelength, emission type, size, weight, and others [4].

For the lidar to receive back its transmitted signal when the reflected light returns, it must contain a photodetector, whose task is to convert an optical into an electrical signal based on the photoelectric effect. The most critical parameter of the detector is expressed in the photosensitivity when receiving photons from the environment, and another essential parameter is the detection of short pulses, which requires a large bandwidth [1,4]. Some of the most used detectors are PIN photodiodes, single-photon avalanche photodiodes (SPADs), avalanche photodiodes (APDs), silicon photomultipliers (SiPMs), and photomultiplier tubes (PMTs) [1,3,4]. Silicon detectors are useful for the 300–1100 nm range, while for wavelengths above 1100 nm, InGaAs or InP detectors are used [1,4]. Besides light transmitter and detector, a typical lidar system also includes a time-to-digital converter (TDC) and signal processing units.

Since lidar and radar both use electromagnetic waves for detection purposes, they are often compared to each other. The shorter wavelength of lidar optical waves results in better resolution, i.e., more precise detection, making it a better choice for creating 3D images and maps. The ability to integrate lidars on an electronic or photonic chip can reduce the size, cost, and power consumption, making it affordable for many applications, including object detection.

### 2.1. Types of Lidar Systems

There are currently several types of lidar systems on the market, which depend on different types of scanners. The scanner positions the laser beam in a particular direction by changing the angle and characteristics of the radiation beam to generate a 3D point cloud, i.e., to collect data for mapping the environment [4]. The specific method of directing the laser beam affects the precision, speed, FOV, and their effect on the object detection, thus directly affecting the obtained image resolution [4]. We distinguish two main systems according to how the radiation beam is directed—mechanical lidar and solid-state lidar [1,4].

In the transportation sector, lidar is most often used for autonomous vehicles, where three types of systems stand out as the best solution. The first solution is a mechanical lidar that uses rotating mirrors and galvanometric or piezoelectric positioning of mirrors and prisms to scan the environment [4]. The other two solutions include solid-state lidar versions—a micro-electromechanical system (MEMS) that uses electromagnet or piezoelectric-controlled micro-mirrors and optical phased arrays (OPAs) that direct a beam of radiation from an array of optical antennas [1,4].

#### 2.1.1. Mechanical Lidar

Mechanical lidar uses expensive optics and a rotating or galvanometric system, with mirrors or prisms attached to mechanical actuators, to provide a wide FOV of usually 360° [4]. Units that include both sources and detectors rotate around the same axis, which can be achieved either by rotating the optical setup around a mechanical axis where several detectors are positioned in parallel along the spinning axis, or by successively aiming the beam across the target in the two-dimensional (2D) space [4]. The rotation of the optical setup is usually the most preferred scanning option because it offers angularly equispaced concentric data lines, or straight and parallel scan lines, with a constant scanning speed over a wide FOV [4,5].

Mechanical lidar structure allows for a large signal-to-noise ratio (SNR) for a wide FOV but with several drawbacks, including larger size, bulky design, high cost, reliability and maintenance issues, and sensitivity to vibrations [1,4]. Mechanical lidar almost always utilizes a pulsed laser source. Moreover, it is characterized by high power consumption and frequency limited to approximately 100 Hz due to the inertia of the rotating assembly [4]. Nevertheless, mechanical lidar is very efficient for long-distance detection (beyond 1 km) and valuable for researching and testing autonomous vehicle systems, algorithm training, and robotics [4]. However, it is not tempting to the end-user due to the abovementioned shortcomings, so it is often replaced by a solid-state version.

#### 2.1.2. Solid-State Lidar

Unlike mechanical, solid-state lidar has no moving mechanical components, resulting in a reduced FOV [1,4]. However, these systems increase their FOV to the one competitive with mechanical lidars by using multiple sensors on each vehicle side and fusing their data [4,6,7]. Solid-state lidars have a higher resolution, are faster, more robust, and cheaper than the mechanical lidar, in addition to being physically smaller and lighter.

The sensor principle of MEMS lidar operation is based on the MEMS mirror embedded in the silicon chip and rotated by balancing the electromagnetic force produced by the coil around it and the elastic force of a torsion bar representing a fixed axis around which it rotates [1,5]. MEMS provides programmable laser beam control using small mirrors whose angle is determined by applying a stimulus, thus directing a beam to a specific location [4]. Depending on the application and other requirements, different electrostatic, magnetic, piezoelectric, and thermal technologies are used for actuation [4]. The most common approach involves controlling the mirrors by drive voltages generated from a memory-stored digital scan pattern using a digital-to-analog converter (DAC) [4]. For MEMS systems, the receiver light collection aperture, defining the receiver SNR, is often fairly small (in the range of a few millimeters) [7]. Lightweight characteristic, compact design, and low power consumption have increased interest in MEMS lidars, so it is increasingly used in the automotive and transportation sectors, as well as in robotics and space exploration [4].

Another representative of the solid-state lidar structure is a novel technology called OPA that, like the previously mentioned MEMS structure, does not use moving components but is based on steering the laser beam using optical phase modulators and multiple micro-structured waveguides [4]. Namely, the speed at which light travels through the device is adjusted using an optical phase modulator, which allows controlling the orientation and shape of the optical wave-front coming from the combined emission of the synced waveguides [4,7]. For example, the beams are delayed by increasing amounts, which allows steering the beam in different directions [7]. This type of lidar system can provide very stable, precise, and rapid beam steering [4]. Moreover, the OPA structure is highly compact, robust, capable of very high measurement speeds of over 100 kHz for a wide FOV, and can be implemented in a single chip, thus gaining interest as a technology with great potential in automotive research and industry [4]. Nevertheless, the laser output power loss is a major disadvantage, so scanning at larger distances is still not feasible [4,8].

### 2.2. Measurement Principles

Given the operating mode of the laser, the lidar can be classified into continuous wave architectures, with the possibility of intensity modulation and pulsating mode architecture where light radiation is emitted by intense short-term pulses. The most popular solutions are pulsed laser, amplitude modulated continuous wave (AMCW) laser, and frequency modulated continuous wave (FMCW) laser.

#### 2.2.1. Pulsed Lidar

In order to determine the distance of an object with a pulsed laser, the travel time of a short-term pulse from the transmitter to the detector is measured. It is desirable that the short-term pulses have as much power as allowed, taking into account the possible exposure and danger to human eyes [4]. Pulses lasting from 1 to 10 ns are used for most applications [9]. Pulsed lasers often use the abovementioned SPAD for detection due to their higher sensitivity and increased operating range, but these photodetectors require a long recovery time and are sensitive to the thermal noise of electrons. The main advantages of pulsed lasers are the simple principle of operation and the possibility of measuring at greater distances [4]. On the other hand, their operation is limited by the SNR of the measurements that requires utilizing intense light pulses while maintaining eye safety limitations and using very sensitive detectors that may significantly increase the cost [4]. Moreover, the utilized electronics have become significantly more sophisticated due to detection requirements in the form of high-frequency rates and large amplification factors [4]. However, this architecture is most commonly used in commercial lidar systems for autonomous driving and transportation, due to its simplicity and ability to work outdoors [4].

#### 2.2.2. AMCW Lidar

AMCW lasers use a continuous light signal of modulated intensity [9]. They use a phase shift to determine the distance of the detected object, which can be considered an indirect TOF measurement. Furthermore, modulation is performed on a constant frequency signal, typically in the range of about 10 MHz on sine or square waveform [4]. AMCW laser does not provide high range resolution, although the accuracy can range within a few centimeters and even less, which satisfies most applications [9]. As laser diodes are often used instead of lasers in the AMCW lidar, the emitting power is limited, which introduces more detection noise and reduces the range, thus limiting the application to interiors such as large object detection or driver and front passenger detection [4].

#### 2.2.3. FMCW Lidar

The FMCW lidar operation depends on the characteristics of the coherent radiated wave [9]. Namely, the source’s power is typically varied to periodically shift the emitted instantaneous optical frequency [4]. By converting the received signal into an optical domain, the need for wideband electrical circuits is eliminated, so it is possible to use traditional complementary metal-oxide semiconductor (CMOS) electronics, which achieve significantly higher range resolution and precision [9]. The sawtooth wave is modulated periodically, and the reflected signal is mixed with the emitting one, thus creating a frequency difference based on which the object’s distance is determined [4,10]. The FMCW method allows the range resolution in millimeters and even less [4]. Due to their speed, they are suitable for use in autonomous vehicles and dynamic traffic detections. The FMCW laser has the highest precision for shorter distances compared to the other operating modes [4]. The problem with the FMCW method is the stability of coherent light within the entire measurement cycle, which arises from the laser characteristics such as temperature stability, precision, voltage linearity dependence, and external conditions [4].

### 2.3. Parameters

The most important parameters of the lidar system are axial precision, FOV, angular resolution, transmitting power with respect to the safety regulations for the human eye, sensitivity to interference and ambient light, maximum operating range, power consumption, and price [9].

Axial or range precision represents the standard deviation of several measurements performed for a target at a fixed distance [9]. It is affected by the distance and target surface properties. Precision is also called a measure of repeatability, and it is necessary to distinguish it from accuracy. Namely, the term accuracy refers to how closely the measured value matches the actual value. Moreover, range resolution represents the system’s capacity to distinguish two or more different closely spaced objects in the axial direction [9]. It is determined by the detected object’s type and size, the transmitted pulse width, and the receiver efficiency. The pulsed lidars provide the centimeter-level resolutions for a wide range of detection distances, including long-distance measurements [4]. Moreover, the AMCW lidars can offer similar precision but only at moderate distances [4]. Finally, the FMCW lidars significantly outperform the other two approaches in terms of achieved resolution by allowing even the micrometer-level resolutions [4].

The FOV is the angle covered by the lidar sensor. Depending on the structure and technology of the particular lidar system, this angle may vary. For example, a rotating lidar uses a mechanical system that rotates the laser through 360° of the environment, whereas less complex systems use fewer lasers and scan sector by sector, directing the laser towards the desired area. Moreover, different angles are used for different scanning applications. The FOV is typically defined by two horizontal and vertical angles around an axis perpendicular to the front of the sensor, within which distance measurements can be performed [9]. Furthermore, angular resolution defines the ability to resolve two adjacent points in the FOV, where optical waves with micrometer wavelength achieve angular resolutions on the order of 0.1°, while requiring aperture sizes in the range of only a few hundred micrometers [7,9]. The FOV and angular resolution of the classic pulsed lidar are determined by the receiver’s optical characteristics and the photodetector’s size, whereas in the beam-steering lidar, the beam properties have a significant impact on these parameters [9]. In addition to using mechanical rotating assembly, the often desired 360° FOV can be achieved computationally by fusing data from multiple sensors [9].

Emission power is defined as the highest energy density of light radiation. Although the higher power of the laser beam is desirable to enable detection at greater distances, its maximum value is limited due to the potential damage to the human eye [9]. Namely, even lasers with milliwatt-level power can cause significant damage [9]. Thus, the maximum permissible exposure (MPE), together with the emitted power, is strictly determined by the wavelength and laser beam diameter, as well as the duration of exposure for continuous mode lasers, and pulse width and frequency for pulsed lasers [9].

The transmitter’s power and the receiver’s sensitivity are generally the two factors that restrict the maximum operating range [9]. Lidar systems can generally detect objects from a few meters to over 200 m away [7]. With more powerful radiation, the operating range increases but requires the use of a larger receiver aperture [9]. Furthermore, the use of beam-steering lidars for long-range applications is a better choice than the classic pulsed lidar considering that the laser power focuses on one point at a time, creating a stronger feedback signal [9].

To illustrate with examples of the values of the parameters described above, the examples of several commercial lidar systems from different manufacturers used in today’s applications are provided next. Velodyne Lidar’s Puck is a small and compact 16-channel lidar that generates up to 600,000 points per second and has a measurement range of 100 m, a range accuracy up to ±3 cm, a 360° horizontal FOV with a 0.1–0.4° horizontal angular resolution, a 30° vertical FOV with a 2.0° vertical angular resolution, a 5–20 Hz rotation rate, and power consumption of 8 W [11]. Moreover, Velodyne Lidar’s Alpha Prime is a 128-channel lidar system designed for long-range detection in autonomous mobility, which generates up to 4.6 million points per second and has a measurement range up to 300 m, a range accuracy of ±3 cm, a 360° horizontal FOV with a 0.1–0.4° horizontal angular resolution, a 40° vertical FOV with a minimum vertical angular resolution of 0.11°, a 5–20 Hz rotation rate, and power consumption of 23 W [12]. Furthermore, RIEGL’s VUX-1HA is a kinematic lidar that generates up to 1.8 million points per second and has a maximum measurement range of 475 m (depending on the target’s reflectivity and laser pulse repetition rate), a minimum range of 1 m, an accuracy of 5 mm, a precision of 3 mm, a 360° horizontal FOV, an angular resolution of 0.001°, and power consumption of 65 W [13]. Finally, Leica ScanStation P50 is a long-range terrestrial lidar that generates up to 1 million points per second and has a maximum measurement range greater than 1 km (for 80% target’s reflectivity), a minimum range of 0.4 m, a range accuracy of 1.2–3 mm, a 360° horizontal FOV, a 290° vertical FOV, and a horizontal and vertical angular accuracy of 8″ [14].

## 3. Application of Lidar System in Road Transportation

Due to the previously analyzed characteristics of lidar systems of high precision and resolution, the technology has found application in the transportation sector, both in autonomous vehicles for detection of obstacles, pedestrians, roads, and other vehicles, and in other traffic systems for recognizing objects on the road and along the road [15].

A key factor in using the lidar system in transportation is generating a 3D image, called a point cloud, representing a computer representation of the actual situation in the vehicle’s environment. After lidar scanning, a 3D grid of data points is provided where the data files obtained by measurements contain information on the distance of each detected point in 3D space defined by the X-Y-Z coordinates, as well as the information on the reflectivity data over time [2]. Different colors in these 3D representations indicate the intensities of the radiation energy reflected from particular points. Any location within this computer-generated 3D scenery can be selected as the observer’s point of view [2]. The computer analyzes a vast number of these points in real-time using the visual information from multiple views to assess the environmental conditions [2]. Neighboring points moving together are detected, recognized, and classified as specific objects [2]. The obtained 3D lidar images can help us plan, simulate, map, and visualize situations, with the possibility of training decision-making algorithms.

**Figure 2:**
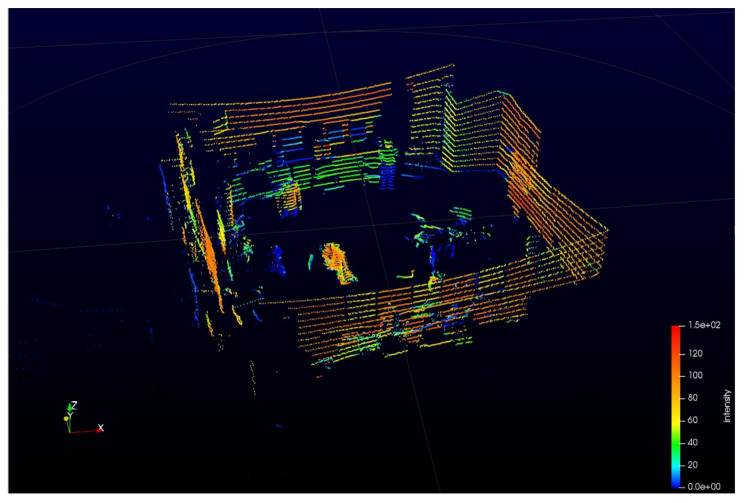
Example of 3D point cloud representation obtained by the lidar system.

An example of such lidar-generated 3D representation, i.e., point cloud, is shown in Figure 2. This image shows the laboratory interior, including several people and laboratory equipment. It was obtained using Velodyne’s Puck lidar [11,16], which utilizes an array of 16 infrared lasers connected to infrared detectors.

Mobile laser scanning (MLS) is a system that uses laser scanner technology to create highly reliable 3D images that can be used for a wide range of needs [17]. The system consists of a 3D laser scanner that measures the distance of detected objects from the device, the Global Navigation Satellite System (GNSS) that determines and saves the position, an inertial measurement unit (IMU) that determines the correlation of position between data and photo or video camera that captures the color spectrum of the radiation from the recorded objects [17,18]. The components are usually mounted on a vehicle or person moving in the area where the scan is taking place [17]. Thanks to the technology discussed in the previous section, MLS systems are able to measure very small details, even at the millimeter level, with high point density [17]. The collected data are often used in classification algorithms for machine learning and prediction developed in the fields of sensors, robotics, and computer vision [17]. In the last decade, the intensity of the research and development of MLS systems has increased significantly.

The main advantage of the MLS system is high precision and measurement resolution that can range around several thousand measured points per square meter, with a centimeter or even millimeter precision [17]. For comparison, the data density measured by the airborne laser scanning (ALS) technique usually ranges up to 10 points per square meter, and the typical resolution of individual points ranges between 30 and 50 cm [17]. Although the density of measurements with the terrestrial laser scanning (TLS) technique can reach the same level as MLS, measurements are often performed without an IMU, so individual measurements from different positions are more difficult to combine into a single image [17]. On the other hand, the disadvantage of the MLS system is the very high measurement density that causes large files within gigabytes for each recorded kilometer, which results in lengthy image processing and image generation time [17]. Furthermore, MLS cannot perform fine-detail measurements due to the urban environment’s terrain characteristics [17]. Therefore, in urban environments consisting of natural and man-made elements, a combination of MLS and ALS techniques is used to create a complete picture [17]. However, due to the mentioned significant progress in surveying techniques, a detailed survey with the MLS technique is sufficient in many cases, from which road markings, pavement edges, pedestrian crossings, trees, street poles, traffic signs, traffic lights, and buildings can be distinguished.

The collected MLS system data consist of distances and angles, along with timestamps that reference the exact position relative to the measuring device [18]. Combining vehicle odometry, GNSS, and IMU sensors, the measured data is converted into three-dimensional coordinates to create a point cloud that seeks to recreate the most accurate representation of the scanned area [18]. Most MLS data go through the following processing order: georeferencing data, mapping color information, filtering data, and generating models or extracting features from the point clouds [18].

Additional steps are possible depending on the place of use and the desired result. In order to manage the data collection process with the MLS method, a good knowledge of the system is required for the data processing to give the best possible result [18]. Data georeferencing seeks to combine data from multiple sensors into one complete dataset of a 3D coordinate system with as few errors as possible [18]. As mentioned earlier, lidar, GNSS, and IMU synergy is essential for proper integration. While the scanning system collects distance and location data, a photo or video camera captures colors in the red-green-blue (RGB) spectrum for each measured point in a point cloud, and stores it as a numerical value in the range of 0–255 [18,19]. Then, the colors are mapped by connecting them to the known recorded X-Y-Z coordinates, so each point is characterized by seven descriptive elements (X, Y, Z, R, G, B, I), where I represents the intensity [18]. Color mapping allows a significantly more straightforward method for classifying objects due to their different reflected light. Furthermore, after data recording, filtering is performed to eliminate the data that is not useful for the end result, such as transient objects targeted by the scanner, unwanted vegetation, and the like [18]. Also, filtering is often performed to reduce the size of recorded files because they often take up much space and require a lot of processing power [18]. Filtering is beneficial when measuring on a bendy road or in the middle of an intersection, where the measured data is very dense on the side the operator is turning, and sparse on the opposite side [18].

As MLS technology has brought many advantages over the traditional and static TLS method, its use has led to many benefits for the transportation sector, such as increased safety, efficiency, accuracy, technical improvements, and lower cost [18].

Most commercial MLS systems are based on the already explained TOF method, which results in an extended range, while TLS systems use a phase shift whose features include a higher precision and a higher density of scanned points [20]. Velodyne, Innoviz, Leica, Optech Lynx, LeddarTech, RIEGL, Sense Photonics, DYNASCAN MDL, Blickfeld, Trimble, and SICK are examples of TOF-based scanners, whereas Z+F and FARO scanners stand out among phase shift-based TLS systems [20].

**Figure 3:**
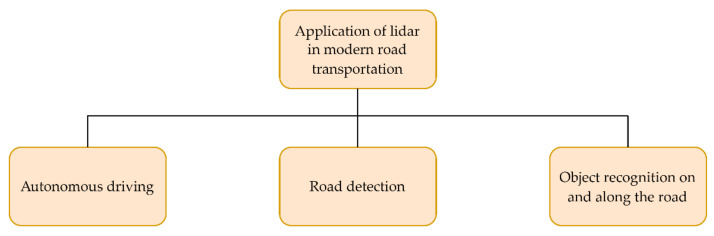
Application of the lidar system in modern road transportation.

Figure 3 shows the flowchart depicting the main areas of application of lidar systems in modern road transportation, which are discussed in this paper. These application areas include autonomous driving, road detection, and object recognition on and along the road. Moreover, an overview of the recent scientific papers on the application and analysis of the lidar system in modern road transportation, which are mentioned and described in this paper, can be found in Table 1. This table briefly summarizes the main application studied and the main findings of each paper.

### 3.1. Application in Autonomous Driving

As MLS data can be used to observe street environments and detect and pinpoint objects, vehicle orientation, and behavior, it can be concluded that the system is crucial for Advanced Driver Assistance Systems (ADAS) and visual perception in autonomous vehicles [2,61]. The most important factors of the MLS system in autonomous driving are the detection of vehicles and pedestrians, the detection of traffic lanes, and the detection of the road or other driving surface [22]. Existing MLS systems are often combined with various cameras and radar sensors [2,62], thus improving the timely detection and prediction of both pedestrians and vehicle movements in traffic. Each of these sensors has its own advantages and drawbacks. Namely, optical cameras are affected by environmental factors, such as lighting, weather changes, and different obstacles obscuring sight, whereas lidar is affected by adverse weather conditions [62]. On the other hand, radar is not affected by environmental light or weather conditions, but has lower detection accuracy and resolution [62]. Therefore, data fusion from these complementary sensors is beneficial for autonomous driving applications [62].

The envisaged autonomous driving system should cover 360° of the environment, not just the isolated sections in front of and behind the vehicle. For higher autonomy, the sensors should adequately detect and expect hazards from the sides. Lateral detection is essential to avoid accidents caused by changing lanes, or to detect other vehicles at intersections.

The successful detection of all obstacles and traffic participants requires the synergy of many sensors that do not only include the lidar system. Thus, various sensors are used depending on the manufacturer and the detection method, such as long-range and short-range radar, ultrasonic sensors, optical cameras, and lidar sensors [21]. Lidar sensors in the range of about 900 nm are limited in terms of distance as opposed to those at 1550 nm, capable of detecting at 200–300 m [21]. In addition to high-resolution detection, which allows the observation of smaller detected details, the lidar is able to measure the speed of objects directly [21]. Likewise, as previously stated for detecting moving objects, vehicles, and other road users, the lidar can also map or record a static environment, which can also serve as a reference when predicting danger [21]. Of all these sensors, lidar is the most efficient. Working together with a camera, GNSS, and IMU system brings the application to a whole new level. In the future, it is expected that lidar, together with its integrated radar and camera systems, will replace all other sensors, which no other system is capable of. However, the current limit is an enormous amount of data to be processed and a high price that should decrease with technology development over time [21]. Moreover, the development of MEMS lidar could make this system more affordable for autonomous vehicles [5].

The synergy of all sensors helps the computer make decisions for the vehicle’s autonomous driving, be it a system of braking, turning, acceleration, or signaling. Until recently, research on autonomous vehicles, while driving or standing still in a real urban environment, showed a pedestrian detection ability of 93–99%, while the classification of road users (such as cars, cyclists, pedestrians, and trucks) using convolutional neural networks (CNNs) achieves an average accuracy of 97% [17].

Perceptual algorithms are usually trained to detect moving and stationary objects and estimate the ground surface by intentionally ignoring the effects of weather conditions to reduce potential misdetections [24]. However, if lidar systems are planned to be used at higher autonomy levels, it is necessary to observe their behavior even in bad weather conditions such as heavy rain or thick fog [63]. In addition to rain and fog, direct sunlight, smoke, and dirt can also be an obstacle.

This phenomenon is due to the different interactions of light and matter. Namely, light reflection from a surface or object, and detection of such reflected signal, occurs due to various interactions between the radiated signal and matter depending on the wavelength of radiation, the size of the matter, and other causes. When electromagnetic radiation interacts with matter in the form of a molecule or atom, scattering occurs, i.e., a change in the radiated signal’s direction [3]. If the photon changes its direction several times, a multiple scattering process occurs [3]. Moreover, reflection occurs when the direction of movement of radiated electromagnetic waves between two media changes where the signal returns to the medium from which it came. Furthermore, absorption occurs when electromagnetic radiation interacts with matter resulting in the absorbed photon, causing a change in the energy state of the atom or molecule in the matter, i.e., the radiation is converted into heat or some other form of energy [3]. Additionally, fluorescence occurs when a photon is absorbed, and a molecule emits a photon of the same or greater wavelength after a certain time [3]. Finally, the relative motion of the transmitter and detector leads to the Doppler effect manifesting in the radiated waves’ apparent frequency or wavelength shift [3].

When testing the lidar system in demanding conditions, it was proven that dust particles in the air prevented the detection of objects behind them [25]. This was due to the previously explained multiple scattering, reflections, and light absorption. Therefore, an algorithm was developed to reject signals reflected from dust particles by filtering data according to radar. Fog conditions have a similar effect on lidar performance [26,27]. In [28], an algorithm based on the Kalman filter and nearby point cloud denoising was developed to improve the reconstruction of 3D lidar measurements from autonomous vehicles in adverse weather conditions. Further research has shown that radar sensors are generally more robust than lidar sensors and cameras, which are heavily affected by rain and fog. Due to such performance, the distance of detectable objects is drastically reduced.

Furthermore, interesting results were obtained in [29] when analyzing the results obtained by lasers with 905 nm and 1550 nm wavelengths. The target comparison area was high humidity, rain, fog, and wet object detection performance. If the detected object was dry, it generally reflected light better at a wavelength of 1550 nm than at 905 nm (with some exceptions, such as military uniforms). However, the comparison results under adverse conditions showed that the laser at a wavelength of 905 nm retained its nominal characteristics much better than that at 1550 nm. The most considerable difference was observed in rainy conditions (25 mm/h), where the 905 nm laser was able to detect twice the distance than the laser at 1550 nm. The fog had the most significant impact on reducing the detected distance for both cases, yet the 905 nm laser detected up to 60% greater distance than its competitor. The wetness of the objects was not a significant factor in determining the distance, where the leading laser was again more successful by 10–15%. When tested, the humidity did not show any noticeable effect for both wavelengths, which does not mean that it would not be the case when measuring very long (kilometer) distances, where it is expected to impact the results significantly. In addition to the achieved results, it was noted that the laser at a wavelength of 1550 nm is safer for human vision, which can be an essential factor in some applications, given that the 905 nm laser requires more power. Also, 905 nm laser radiation is visible with most night vision systems, so its use for military purposes is not acceptable. Thus, the research in [29] proved a significant difference in the performance of two lasers under the same adverse weather conditions, and recommended using lasers at 905 nm if such conditions are involved.

### 3.2. Road Detection

The use of optical cameras to detect roads and road edges is not an ideal solution due to the high dependence on outdoor lighting. As an alternative, a lidar system is offered that emits its own light, which eliminates dependence on external conditions, so detection is also possible at night. Due to this characteristic, the lidar-based road detection system has the same accuracy level either during the day or at night, achieving satisfactory results for automated vehicles. Several road detection algorithms have been proposed exclusively for data captured by lidar, or fusion of optical camera and lidar data [30], but no solution has long been able to outperform the results of the best optical camera-based system.

The continuation of the research proposed a system based on deep learning which used fully convolutional neural networks (FCNs) [30]. It was tested on the classic Karlsruhe Institute of Technology and Toyota Technological Institute (KITTI) dataset [64], often used for robotics and autonomous driving tests. The newly created Lidar-only Deep Neural Network (LoDNN) system achieved excellent performance, surpassing the previously best lidar-based system by 7.4%. Also, its calculation time was significantly less than one of all other systems, which made it suitable for predicting in real situations, and it showed excellent precision in detecting the edges of the road surface. The potential difficulties encountered by the system were highlighted when detecting connecting sidewalks and pavements, as is the case with pedestrian crossings or when changing terrain elevation. In addition, the FCN provided the best results at a distance of up to 31 m, after which a significant degradation was observed up to a maximum of 46 m. It was concluded that the use of such a system, together with the graphics processing unit (GPU)-accelerated hardware, met the needs of real-time road detection for autonomous vehicles, which did not include distinguishing traffic lanes and their directions. The problem of automatic lane identification was addressed in [31] using the vehicle trajectory data acquired by roadside lidar sensors, achieving satisfactory results even on non-straight roads and with the presence of pedestrians, but failing at intersections.

Building on previous research, a new LidCamNet detection system based on the principle of fusion of data recorded by lidar, and those recorded by the optical camera, was presented in [32]. The FCN fusion concept provided excellent results, achieving an accuracy of 96.03%. Additionally, a continuation of the research was proposed to extend the testing to roads covered with mud or snow to confirm the method’s effectiveness. The study in [33] proposed a road detection and parametrization approach using the lidar data as input to the integrated system, based on the building information modeling (BIM) and geographic information system (GIS). Furthermore, the lidar-histogram method was developed in [34] to detect roads and different obstacles, where the detection problem was reduced to the linear classification in 2D space, as a 3D road plane was projected as a straight line segment with obstacles projected above or below this line.

With the further development of lidar technology, edge detection accuracy has improved, as indicated by the 2018 study where test datasets collected by an autonomous vehicle from Tongji University in China were used [35]. Namely, the “sliding beam” method was utilized, as well as the pavement edge search method, after each taken shot. Processing time with such methods was extremely fast and amounted to 12 ms. However, the average error in determining the edge of the pavement was 32.42 cm, which was not ideal. In addition, it should be noted that in over 90% of cases, the detected pavement edge had a deviation within 10 cm, which means that a large average error was affected by a small number of false-positive results that had a greater distance. Some of these errors were caused by damage to the curbs and other edges. Detailed testing with datasets and real-time detections showed good precision and a robust base for autonomous driving needs, achieving an average accuracy of approximately 85%. Moreover, an automated algorithm for detecting road edges from terrestrial mobile lidar data was proposed in [36] based on the modification of the parametric active contour model. The algorithm was tested on various road types, achieving satisfactory results. However, it is very important to emphasize that detecting road edges is still a significant problem if other vehicles or pedestrians are on the road, which is a common situation.

### 3.3. Object Recognition on the Road and along the Road

In order to correctly determine the position and identify objects in traffic, it is necessary to determine one’s own position well because it serves as a reference for everyone else’s. Solutions using GNSS devices are not acceptable due to frequent errors and unreliability of the satellite signal, while previously created maps are unsuitable because they do not consider the dynamic factor of traffic and environment. Therefore, determining the position in relation to the environment, and recognizing objects on the road and along the road, is often done using systems based on optical cameras and lidars. Other than using a lidar system, it is possible to do the same in a cheaper way using maps and monocular cameras [37]. Some approaches also combine lidar-based localization with GNSS information [38], or utilize GNSS, IMU, and lidar sensor integration for map creation and vehicle navigation [39].

The lidar-based system can determine the position of the detected object and plan the trajectory according to the vehicle position information. This improves the autonomous vehicle’s functionality due to the technique’s accuracy and precision. Due to the already explained unreliability of satellite systems and objects that block the signal, such as buildings, trees, and tunnels, the GNSS system is impractical, and map-based localization is required. The data from the sensors are combined with the maps, and due to their synergy, the possible position of the vehicle is estimated. Environmental features that include roads, road edges, buildings, and traffic signs are often used as a reference to connect data and thus determine the distance between objects and vehicles [22]. Instead of optical cameras that capture environmental features, a robust localization system based on a multilayer lidar that is immutable with respect to external light was proposed in [40]. The study’s success was manifested by 14–27 cm longitudinal and transverse distance errors, even in the presence of other vehicles in traffic. Furthermore, the system’s accuracy over certain datasets reached value, i.e., average error, within 15 cm.

The study in [41] proposed a vehicle localization approach based on the free-resolution probability distributions map (FRPDM) generated by Gaussian mixture modeling (GMM) using 3D lidar data, allowing efficient object representations, smaller map sizes, and good position and heading estimation accuracy in the tested urban area. Moreover, the authors in [42] approached the vehicle pose estimation problem by utilizing the lidar data and ensemble learning network trained on the time series and spatial tightness evaluation indexes, improving estimation accuracy, even at curved road segments. Furthermore, the autonomous vehicle localization method based on the IMU, wheel encoder, and lidar odometry was presented in [43] and provided accurate and high-frequency results in a diverse environment.

Using a monocular camera as a competitive and cheaper method has shown results that, although not superior, compete with results based on the lidar system, especially when looking at the performance-price ratio [37].

Recognizing road markings and traffic signs near the vehicle is a crucial task for higher levels of autonomy, where the vehicle alone should perform most, if not all, necessary actions, and a useful factor at lower levels of autonomy to warn or inform the driver. As for other objects detected by the lidar, the traffic signs must be extracted from the created point cloud. The method of interpretation and extraction of points will depend on the desired object, the application type, and the object’s geometric characteristics [44]. Shape and pattern can play a significant role in interpreting points and are especially useful in detecting man-made objects, such as traffic signs [44]. In general, pavement markings consist of a set of predefined shapes that can be combined with each other (e.g., rectangle, dashed line) [44]. In many cases, the markings have linear characteristics and previously known dimensions [44].

Recent studies have approached pavement recognition by converting 3D to 2D georeferenced images, using weighted neighboring difference (WND) histograms, inverse distance weighted (IDW) data interpolation, and the multiscale tensor voting (MSTV) method [45]. Data were collected by the commercial RIEGL VMX-450 mobile lidar system [65], and the results proved to be much better than the previously considered studies, with an accuracy of about 90%. Also, the 2014 study at the University of the Czech Republic proposed a way to recognize road markings, traffic signs, fences, and other structures using two data sources [46]. The data collected from the camera and the lidar sensor were used together to determine the objects. Like the previously mentioned study, the RIEGL VMX mobile lidar system was used and applied in the urban area. The success rate of detecting traffic signs was 93%, noting that the method was very successful in detecting an independent traffic sign. However, this method could not distinguish a single sign if multiple signs were located on a single structure. If detection was attempted with predetermined datasets that included known traffic signs, a very good accuracy of over 90% could be expected [47]. Namely, in the study conducted in the USA with a predetermined set of 112 different signs and using the weakly supervised metric learning (WSMLR) method, the camera and lidar sensor data were also used together. Individual tests resulted in an accuracy of over 95%, which was satisfactory for the initial phase of the research. Furthermore, in [48], an automatic highway sign extraction method was developed based on applying multiple filters to point cloud data and performing clustering. The experimental tests showed high accuracy of this fast and straightforward method, with higher efficiency for road segments without overhead signs. The results indicated the method’s potential to generate traffic sign inventory, or import these data into autonomous vehicle applications.

It is evident from the presented studies that the success of detecting road markings and traffic signs is very satisfactory during real-life experiments, as well as those using available datasets. However, although the results are presented as very good, most, if not all, systems are not ready to be used in everyday conditions. If not indicated otherwise, most systems are tested in good weather conditions, which does not provide system performance data when it rains or fog forms.

The autonomous vehicle perceives the environment with its sensors and determines the importance and significance of particular objects. The process that the lidar system performs over the collected data usually consists of the following four steps: object detection, object recognition, object tracking, and motion prediction [1]. This four-step division is acceptable for popular mechanical lidars, such as the already mentioned Velodyne lidars, where the processing is done in spherical coordinates (r, φ, θ) [1]. Object detection involves estimating their physical characteristics, position, and shape [1]. As a first step, it also involves initial filtering and clustering [1]. Detection, and thus object recognition, can also be performed by machine learning, which provides the classic categories (such as pedestrian, car, truck, tree, and building) [1]. The data features are extracted, and the data are classified based on them [1]. Tracking objects involves recording their current state, trajectory, orientation, and speed [1]. Bayesian and various versions of the Kalman filter frameworks are usually used for this purpose [1]. Tracking a single object extends to tracking multiple objects simultaneously with an interacting multiple model (IMM) filter that consists of multiple filters connected in parallel using a different motion model [1]. The fourth step includes predicting behavior beneficial for autonomous vehicles and other autonomous systems, where previously described steps provide the past and current state of the detected objects [1]. For example, the authors in [66] proposed predicting car behavior and movement in traffic by the GMM or hidden Markov model (HMM). However, it is worthwhile for each step to impose machine learning that expands and supplements each step. SemanticKITTI [67] and RangeNet [68] are examples that use deep learning methods that take point clouds and perform the objects classification, segmentation, and prediction [1].

Furthermore, the study in [49] investigated pedestrian and vehicle detection and tracking at intersections using infrastructure-mounted lidar sensors. The roadside lidar data were clustered by the modification of the density-based spatial clustering of applications with noise (DBSCAN) [69] method, and the vehicles and pedestrians were classified by a backpropagation artificial neural network (BP-ANN). Finally, the tracking was conducted by a discrete Kalman filter. The experimental tests showed the accuracy of the proposed approach above 95% and the detection range of about 30 m. Moreover, the roadside lidar data was also used in [50] by developing a method consisting of background filtering, lane identification, and vehicle position and speed tracking, achieving a similar detection range. However, significant improvements and algorithm development were still required to allow accurate vehicle type classification.

In recent years, CNNs, as special types of deep learning algorithms, have achieved state-of-the-art performances in image classification, recognition, and segmentation in various research fields and commercial applications [70,71,72,73,74]. Consequently, recent studies on the application of lidar systems in autonomous vehicles have turned to object detection, classification, and prediction using CNNs. Test data are collected by some of the known commercial lidar systems combined with optical cameras, or using KITTI datasets. There are various applications of CNNs, where some studies use them only for a specific step, such as vehicle detection [51,52], pedestrian detection [53], and object classification [54,55,56,57,58], while some try to use them for the whole process. For example, in [59], the authors proposed an end-to-end (E2E) self-driving algorithm utilizing a CNN that provided the vehicle speed and angle as outputs based on the input camera and 2D lidar data. In addition to using CNNs, the support vector machine (SVM) classifier can also be mentioned, which can be used to detect pedestrians and predict their movements [60].

## 4. Application of the Lidar System in the Maritime Sector

The development of the maritime sector, and increasing traffic on the seas and oceans, is creating an increasingly complex situation, as previously discussed for road transportation. In the last 30 years, the number of ships and other vessels in some areas has increased by over 300%. New technologies are also being applied for maritime transport to function efficiently and safely [75], including lidar systems.

**Figure 4:**
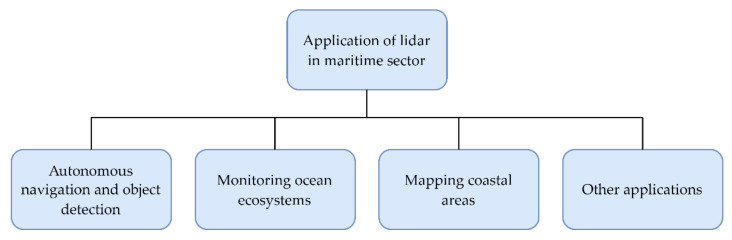
Application of the lidar system in the maritime sector.

The flowchart shown in Figure 4 depicts the main areas of application of lidar systems in the maritime sector, which are discussed in this paper. These application areas include autonomous navigation and object detection on seas and oceans, monitoring ocean ecosystems, mapping coastal areas, and other diverse applications. Moreover, following the same structure, Table 2 provides a brief overview of the recent studies on lidar application in the maritime sector, including application description and main conclusions.

### 4.1. Autonomous Navigation and Object Detection on Seas and Oceans

The development of a lidar system for object detection on the seas and oceans, in addition to greater safety for modern ships, offers the possibility of developing autonomous navigation [76,94,95,96]. Autonomous ships, as well as road vehicles, promise greater safety and lower fuel consumption, which is particularly important today in the context of maritime transport emission reduction [97,98]. The authors in [76] studied the integration of various sensors into a system that should enable the situational awareness of autonomous vessels when combined with artificial intelligence (AI) techniques. The analyzed sensors included absolute positioning sensors (GNSS and IMU), visual sensors (cameras), audio sensors (microphones), and sensors for remote sensing (lidar and radar). Several reasons were detected for the current lidar systems not being widely used for object detection onboard autonomous vessels. One of the discussed drawbacks was the laser power being limited due to eye-safety issues. Moreover, today’s lower-cost commercial lidar systems are intended for automotive applications, thus being focused on cost and size optimization, while having lower operational ranges than those required in maritime navigation of larger vessels. On the other hand, lidar systems used for remote survey purposes have longer ranges, but the required optics are prohibitively expensive and not designed for harsh working environments characteristic of maritime vessels, such as adverse weather conditions and constant motion.

There are several recent studies on utilizing lidar systems in vessel berthing. The study in [77] proposed a berthing information extraction system based on the 3D lidar data and experimentally validated it on the ro-ro ship berthing, demonstrating the effectiveness of the proposed approach in dynamic target recognition and safe ship berthing. Another berthing perception framework for maritime autonomous surface ships based on the shipborne lidar was presented in [78]. The developed procedures for estimating the vessel’s berthing speed, angle, distance, and other parameters from the 3D point cloud lidar data, satisfied the required accuracy for berthing in real-time. Furthermore, the study in [79] described the development of a low-cost lidar-based ship berthing and docking system, proposing a novel method of fusing lidar data with GNSS positioning data. The performance of the proposed system was analyzed during several berthing maneuvers and compared to the performance of the commonly used GNSS-based navigational aid system, proving its usefulness in safe berthing experimentally. Additionally, bollard segmentation and position estimation from lidar point cloud data for autonomous mooring was proposed in [80]. Moreover, the authors in [81] developed a system based on the dual-channel lidar for rotorcraft searching, positioning, tracking, and landing on a ship at sea.

The information received by the lidar system can also be collected using radar, which is often mentioned in the case of detecting objects at sea. Because of that, it is necessary to analyze and understand what improvements the proposed lidar system brings. The already well-known application of radar on ships competes with lidar technology with its ability to detect objects at greater distances and perform better in poor weather conditions, such as rain, fog, or snow [82]. Also, radar sensors are more affordable than lidar. On the other hand, unlike radar, lidar is more precise and detailed when measuring, and can also detect non-metallic objects such as rocks and vessels made of wood or plastic [82]. Additionally, as the lidar system detects fewer objects and noises in the seas and oceans, its task should be simpler than the one required in road traffic [82]. Due to these differences and yet similarities in detection, one system cannot replace another, so a combination of both systems is proposed for the best possible results [82]. Furthermore, a fusion of sensors is required to provide greater safety for all potential conditions, including radar, lidar, cameras, and Global Positioning System (GPS) devices.

The SICK lidar system has been proposed for the application in detecting objects at sea and is characterized by better performance over longer distances and a lower price than its competitors [82]. Some studies conducted measurements [83] and mappings [84] based on the Velodyne lidar system using the SVM classifier, but cannot compete with the SICK lidar in terms of operating range. Lidars with 360° FOV often have a smaller maximum range, while the SICK lidar can detect up to 300 m with an FOV of 85° [82]. Ships, especially autonomous ones, require timely detection of objects because avoiding them is much more difficult and complex than in road traffic, which emphasizes the importance of the operating range factor [82]. Velodyne’s competitive lidar system has the ability to detect with a FOV of 360° and a more detailed recording. However, the SICK system, with a larger operating range and a lower price, is also characterized by a better temperature characteristic [82]. Namely, the SICK lidar operates at a higher temperature range than the Velodyne, giving the proposed system a greater advantage for working in very cold or hot areas [82]. Other competing systems include Quanergy and LeddarTech, which provide similar performance in their respective price ranges [82].

Although there are fewer objects and noises at sea [82,99], measuring with lidar is not that simple. Since light penetrates the water, there will be no feedback signal [82], so the sea cannot be detected the same as the road. The most common stationary objects at sea are rocks and buoys, but light can also be reflected from algae and seagrass floating on the sea’s surface, resulting in a false positive detection [82]. Among other things, the system detects surface sea turbulence caused by the ship’s propeller [82]. Therefore, it is difficult to filter out false-positive data because a rock or similar obstacle may have been detected [82]. Other sensors onboard can also cause some complications [82]. The pulses of the lidar system can interfere with GPS receivers, so the simplest solution is to remove the device from each other’s vicinity [82]. Radio waves from the radar can create noise detected by the lidar, so such a problem is solved by filtering according to the periodic action, i.e., the rotation of the radar system [82]. Among other things, when measuring, the rocking factor of the ship, whether longitudinal or transverse, must be taken into account.

After scanning the environment and collecting data to form point clouds, grouping of points, i.e., clustering, is performed. As with road transportation, several algorithms can be used for clustering, but the most acceptable clustering is often achieved with the DBSCAN method. The method is simple because only two parameters need to be adjusted, including the maximum distance of two points to be considered part of the same object and the minimum number of points needed for something to be considered an object [82]. Despite uneven recognition due to the constant angle between laser beams, the DBSCAN method was selected as the best solution for categorizing objects on lidar datasets in [82].

### 4.2. Monitoring Ocean Ecosystems

In addition to possible applications for detecting objects at sea, lidar systems, if set up accordingly, are suitable for monitoring and analyzing sea and ocean ecosystems. A review of the development of profiling oceanographic lidars was provided in [85]. These remote sensing systems can provide optical properties of the water columns and allow studying distributions of animal species, such as fish and plankton. Moreover, they also provide information on the dynamics of the upper ocean. Furthermore, the authors in [86] described the application of lidar technology in monitoring, mapping, and quantifying coral reef ecosystems. In addition to aerial lidar, satellite lidar can also be used for ocean ecosystem monitoring applications. In [87], an approach combining satellite ocean color observations with the data from a spaceborne lidar was presented, and its application in the study of ocean subsurface properties, especially the plankton properties, was further analyzed.

### 4.3. Mapping Coastal Areas

Lidar ecosystem monitoring provides the potential to use this remote sensing technology for research and mapping coastal areas. Following the monitoring of coastal areas, the detection of sediments on the shores, their deposition, or soil erosion can be mentioned, which can affect changes in temperature and increase the acidity of water.

As one of the most significant applications in the field of geomorphology, lidar is used with GPS to precisely survey coastal areas and create up-to-date topographic and bathymetric coastal maps [88]. Such capability provides an opportunity for many scientific studies, including flood zone delineation, ecosystem protection, monitoring of changes in sandy beaches and other shallow areas after storms or prolonged sedimentary processes, and classification of large water areas [89]. Previous coastal analysis has been based on historical aerial photographs and topographic maps, but topographic and depth data can now be effectively collected by aerial laser imaging with ALS systems, including unmanned aerial vehicle (UAV) lidar [90].

Lidar is most effective when measuring clean seas and oceans where the detected depth reaches up to 50 m. Best water penetration is achieved by using a blue-green laser with a wavelength of about 530 nm [88]. Unlike popular lasers at 1064 nm, its accuracy and range resolution are lower, but it is still a better choice for measuring underwater areas due to the approximately exponential attenuation of energy in water with increasing wavelength [88]. Another interesting application that has become increasingly important recently is detecting and recognizing waste on shores, with a possible classification into plastic, paper, fabric, and metal [91].

### 4.4. Other Applications

In addition to the most prominent application in maritime transport for detecting objects, and the already mentioned monitoring of flora and fauna in waters and mapping coastal areas, lidar is also suitable for many other applications in the maritime sector.

Using signal scattering and reflection as a detection factor also makes it possible to monitor upper ocean dynamics, waves, turbulence, and the impact of offshore wind farms [92]. Nd:YAG laser with the “Q-switching” technique is often used for many of these marine applications due to its reliability [85], while the blue laser would be more suitable for analyzing deeper areas of the sea and ocean up to 100 m, but its application is somewhat prevented by its complexity [88].

Also, Doppler lidar can measure wind speed and direction, which is especially useful for developing and designing offshore wind farms [93]. The Doppler lidar works similarly to the Doppler radar, except that aerosol particles are used for scattering and feedback measurements.

## 5. Challenges and Future Trends

**Figure 5:**
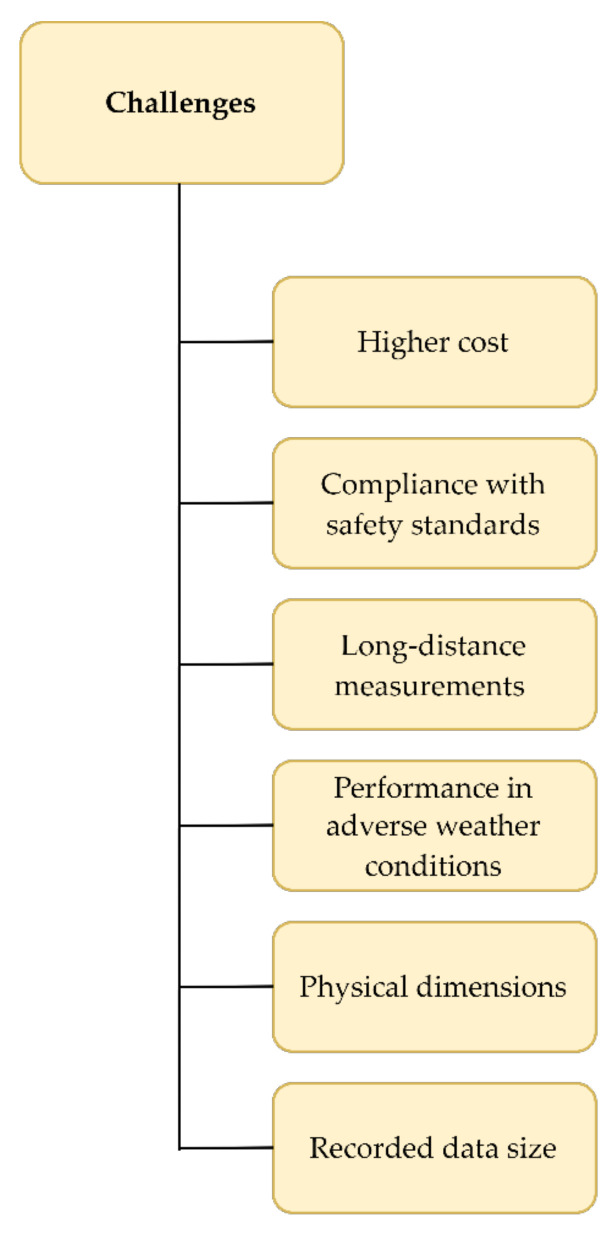
Challenges in lidar system application in modern transportation.

The undeniable fact is that lidar technology finds its applications in ever wider areas and has unique characteristics. However, in today’s systems, several limitations and challenges need to be overcome, such as high cost, compliance with safety and reliability standards, measurements over long distances for highways and maritime needs, performance in adverse weather conditions, and the need for the smaller physical size of the device that encourages integration [1]. As measurements are most often carried out on the move, whether in road or maritime transportation, the measurements are dependent on high-precision GPS and IMU devices for precise georeferencing [18]. The size and complexity of the recorded data also pose a significant challenge [45]. Existing variable laser source solutions (905 nm, 1064 nm, 1550 nm), operating principles (pulsed, AMCW, FMCW), or scanning methods (mechanical, MEMS, OPA) are used to overcome some of the difficulties [1]. A graphical overview of the currently most important challenges in lidar application in modern transportation is provided in Figure 5.

Although it is difficult to predict which type will dominate in the future, lidar use will undoubtedly be introduced in more and more experimental systems for various applications [1]. Existing and future algorithms used to accurately extract and understand data are increasingly showing, and will continue to show, the potential of lidar technology. Moreover, the use of machine learning, and deep learning, in particular, is expected to make even faster progress than that achieved by classical methods, and is considered one of the most important directions of technology in the future. Standardization of control, accuracy, interoperability, and data quality will set guidelines for technology development. The systems are expected to be often integrated with other sensors and developed on different platforms in future applications. Integrating data with other sensors allows the possibility of making conclusions that would not be possible by observing individual data on their own [86], and allows a better understanding of the collected information. The development of the system encourages application in various areas along with transportation, such as geomorphology, ecology, meteorology, marine biology, and many others. It should be mentioned that lidar technology is not expected to replace existing methods but to complement them. Of course, as technology evolves and research reveals new techniques and algorithms, further improvements and expansions of the capabilities and applications of lidar-based systems are expected.

## 6. Conclusions

Although lidar technology was first applied in 1961, it has experienced its most significant development in the last two decades. By observing the results of recent studies and research, it can be concluded that lidar systems provide a vast potential for the development of road and maritime transportation, as well as for related scientific disciplines such as geomorphology, ecology, meteorology, biology, and others. It is evident that the interest in the mobile lidar system in the modern transportation sector is rapidly growing, which encourages the standardization of specific methods and the improvement of models. Interoperability allows flexible data management between multiple technology systems that yield more accurate results and new insights, while the greatest attention is paid to road transportation due to its high complexity and most common application. The technology is characterized by its efficiency, flexibility, speed, size, and precise data collection, creating a 3D point cloud of high resolution. Furthermore, the resistance of semiconductor systems to vibrations enables the detection of objects in a more accessible and reliable way than previous solutions.

An overview of existing and emerging applications shows the system’s characteristics, existing architectures and their limitations, and the challenges for current and future systems. The market currently offers several models from several manufacturers for different purposes that provide satisfactory results, and new models with better technical characteristics are presented every year. Although the technology is not perfect, it is expected to see better performance and wider application across many different areas through further development.

Therefore, future research and development in lidar technology application in modern transportation will focus on addressing the current challenges and technology limitations. This primarily refers to increasing the detection range where a reliable detection of objects at larger distances is necessary for many traffic situations, to allow timely reaction by either human operator or autonomous system. An example of such a situation in road transportation is driving at higher speeds on highways, while maritime transportation and autonomous navigation are generally characterized by maneuvers that take more time to perform. Improved long-distance detection will require technological improvements to the lidar system components, the improved resolution of the 3D point clouds, and further development and upgrades of the utilized computational algorithms.

Machine learning, particularly deep learning, will play a significant role in enabling longer-distance detections and generally more reliable application of lidar technology in transportation. This will include further improvements to the existing algorithms, as well as the development of specific approaches oriented towards 3D lidar data. CNNs have a great potential here as they have been shown to provide state-of-the-art performances in image classification, recognition, and segmentation. Furthermore, the continuous development of the utilized electronics should reduce the lidar system’s dimensions and allow integration with various autonomous systems. Moreover, another area of possible improvement, which will be crucial for the lidar system autonomy and its final beginning of widespread application in real-world transportation systems, is improved performance in adverse weather conditions. This ultimately important area will require the development of highly specialized signal processing algorithms. Finally, novel signal processing approaches will also play a vital role in fusing data from various sensors as lidar will be an integrated part of multi-sensor systems.

## Figures and Tables

**Table 1:** Review of scientific papers on the application and analysis of the lidar system in modern road transportation.

	Reference	Description of Application	Conclusion
**Autonomous driving**	[1]	A review of the state-of-the-art lidar technologies and the associated perception algorithms for application in autonomous driving	The limitations and challenges of the lidar technology are presented, as well as the impressive results of the analyzed algorithms
	[21]	Discussion of the lidar systems’ role in autonomous driving applications	The vital role of monitoring fixed and moving objects in traffic
	[22]	A review of lidar applications in automated extraction of road features and a discussion on challenges and future research	Use of lidar for various transportation applications, including on-road (road surface, lane, and road edge), roadside (traffic signs, objects), and geometric (road cross, vertical alignment, pavement condition, sight distance, vertical clearance) information extraction
	[23]	Simultaneous localization and mapping (SLAM)-based indoor navigation for autonomous vehicles directly based on the three-dimensional (3D) spatial information from the lidar point cloud data	A comparative analysis of different navigation methods is conducted based on extensive experiments in real environments
	[24]	Extensive analysis of automotive lidar performance in adverse weather conditions, such as dense fog and heavy rain	Poor perception and detection of objects during rain and fog; the proposed rain and fog classification method provides satisfactory results
	[25]	Testing the lidar system for outdoor unmanned ground vehicles in adverse weather conditions, including rain, dust, and smoke	Signal attenuation due to scattering, reflection, and absorption of light and the reduction of detection distance are identified
	[26]	Analysis of the effects of fog conditions on the lidar system for visibility distance estimation for autonomous vehicles on roads	The visibility distances obtained by lidar systems are in the same range as those obtained by human observers; the correlation between the decrease in the optical power and the decrease of the visual acuity in fog conditions is established
	[27]	Analysis of the performance of a time-of-flight (ToF) lidar in a fog environment for different fog densities	The relations between the ranging performance and different types of fog are investigated, and a machine learning-based model is developed to predict the minimum fog visibility that allows successful ranging
	[28]	Application of Kalman filter and nearby point cloud denoising to reconstruct lidar measurements from autonomous vehicles in adverse weather conditions, including rain, thick smoke, and their combination	The experiments in the 2 × 2 × 0.6 m space show an improved normal weather 3D signal reconstruction from the lidar data in adverse weather conditions, with a 10–30% improvement
	[29]	Analysis of the influence of adverse environmental factors on the ToF lidar detection range, considering the 905 nm and 1550 nm laser wavelengths	A significant difference in the performance of the two laser types is identified—a 905 nm laser is recommended for poor environmental conditions
**Road** **detection**	[30]	Deep learning road detection based on the simple and fast fully convolutional neural networks (FCNs) using only lidar data, where a top-view representation of point cloud data is considered, thus reducing road detection to a single-scale problem	High accuracy of road segmentation in all lighting conditions accompanied by fast inference suitable for real-time applications
	[31]	Automatic traffic lane detection method based on the roadside lidar data of the vehicle trajectories, where the proposed method consists of background filtering and road boundary identification	Two case studies confirm the method’s ability to detect the boundaries of lanes for curvy roads while not being affected by pedestrians’ presence
	[32]	Deep learning road detection based on the FCNs using camera and lidar data fusion	High system accuracy is achieved by the multimodal approach, in contrast to the poor detection results obtained by using only a camera
	[33]	Road detection based on the lidar data as input to the system integrating the building information modeling (BIM) and geographic information system (GIS)	Accurate road detection is achieved by lidar data classification, but additional manual adjustments are still required
	[34]	Lidar-histogram method for detecting roads and obstacles based on the linear classification of the obstacle projections with respect to the line representing the road	Promising results in urban and off-road environments, with the proposed method being suitable for real-time applications
	[35]	Road-segmentation-based pavement edge detection for autonomous vehicles using 3D lidar sensors	The accuracy, robustness, and fast processing time of the proposed method are demonstrated on the experimental data acquired by a self-driving car
	[36]	An automated algorithm based on the parametric active contour model for detecting road edges from terrestrial mobile lidar data	Tests on various road types show satisfactory results, with dependence on the algorithm parameter settings
**Object** **recognition on and along the road**	[37]	Visual localization of an autonomous vehicle in the urban environment based on a 3D lidar map and a monocular camera	The possibility of using a single monocular camera for the needs of visual localization on a 3D lidar map is confirmed, achieving performance close to the state-of-the-art lidar-only vehicle localization while using a much cheaper sensor
	[38]	Probabilistic localization of an autonomous vehicle combining lidar data with Kalman-filtered Global Navigation Satellite System (GNSS) data	Improved localization with smooth transitions between using GNSS data to using lidar and map data
	[39]	Generating high-definition 3D maps based on the autonomous vehicle sensor data integration, including GNSS, inertial measurement unit (IMU), and lidar	Existing autonomous vehicle sensor systems can be successfully utilized to generate high-resolution maps with a centimeter-level accuracy
	[40]	Vehicle localization consisting of curb detection based on ring compression analysis and least trimmed squares, road marking detection based on road segmentation, and Monte Carlo localization	Experimental tests in urban environments show high detection accuracy with lateral and longitudinal errors of less than 0.3 m
	[41]	Vehicle localization based on the free-resolution probability distributions map (FRPDM) using lidar data	Efficient object representation with reduced map size and good position accuracy in urban areas are achieved
	[42]	Optimal vehicle pose estimation based on the ensemble learning network utilizing spatial tightness and time series obtained from the lidar data	Improved pose estimation accuracy is obtained, even on curved roads
	[43]	Autonomous vehicle localization based on the IMU, wheel encoder, and lidar odometry	Accurate and high-frequency localization results in a diverse environment
	[44]	Automatic recognition of road markings from mobile lidar point clouds	Good performance in recognizing road markings; further research is needed for more complex markings and intersections
	[45]	Development and implementation of a strategy for automatic extraction of road markings from the mobile lidar data based on the two-dimensional (2D) georeferenced feature images, modified inverse distance weighted (IDW) interpolation, weighted neighboring difference histogram (WNDH)-based dynamic thresholding, and multiscale tensor voting (MSTV)	Experimental tests in a subtropical urban environment show more accurate and complete recognition of road markings with fewer errors
	[46]	Automatic detection of traffic signs, road markings, and pole-shaped objects	The experimental tests on the two-kilometer long road in an urban area show that the proposed method is suitable for detecting individual signs, while there are difficulties in distinguishing multiple signs on the same construction
	[47]	Recognition of traffic signs for lidar-equipped vehicles based on the latent structural support vector machine (SVM)-based weakly supervised metric learning (WSMLR) method	Experiments indicate the effectiveness and efficiency of the proposed method, both for the single-view and multi-view sign recognition
	[48]	Automatic highway sign extraction based on the multiple filtering and clustering of the mobile lidar point cloud data	The tests conducted on three different highways show that the proposed straightforward method can achieve high accuracy values and can be efficiently used to create an accurate inventory of traffic signs
	[49]	Pedestrian and vehicle detection and tracking at intersections using roadside lidar data, the density-based spatial clustering of applications with noise (DBSCAN), backpropagation artificial neural network (BP-ANN), and Kalman filter	The experimental tests with a 16-laser lidar show the proposed method’s accuracy above 95% and detection range of about 30 m
	[50]	Vehicle tracking using roadside lidar data and a method consisting of background filtering, lane identification, and vehicle position and speed tracking	Satisfactory vehicle detection and speed tracking in experimental case studies, with a detection range of about 30 m; difficulties in the vehicle type identification
	[51]	Vehicle detection from the Velodyne 64E 3D lidar data using 2D FCN, where the data are transformed to the 2D point maps	An end-to-end (E2E) detection method with excellent performance and a possibility for additional improvements by including more training data and designing deeper networks
	[52]	Convolutional neural network (CNN)-based multimodal vehicle detection using three data modalities from the color camera and 3D lidar (dense-depth map, reflectance map, and red-green-blue (RGB) image)	The proposed data fusion approach provides higher accuracy than the individual modalities for the Karlsruhe Institute of Technology and Toyota Technological Institute (KITTI) dataset
	[53]	Camera and lidar data fusion for pedestrian detection using CNNs, where lidar data features (horizontal disparity, height above ground, and angle) are fused with RGB images	The tests on the KITTI pedestrian detection dataset show that the proposed approach outperforms the one using only camera imagery
	[54]	CNN-based classification of objects using camera and lidar data from autonomous vehicles, where point cloud lidar data are upsampled and converted into the pixel-level depth feature map, which is then fused with the RGB images and fed to the deep CNN	Results obtained on the public dataset support the effectiveness and efficiency of the data fusion and object classification strategies, where the proposed approach outperforms the approach using only RGB or depth data
	[55]	Real-time detection of non-stationary (moving) objects based on the CNN using intensity data in automotive lidar SLAM	It is demonstrated that non-stationary objects can be detected using CNNs trained with the 2D intensity grayscale images in the supervised or unsupervised manner while achieving improved map consistency and localization results
	[56]	Target detection for autonomous vehicles in complex environments based on the dual-modal instance segmentation deep neural network (DM-ISDNN) using camera and lidar data fusion	The experimental results show the robustness and effectiveness of the proposed approach, which outperforms the competitive methods
	[57]	Road segmentation, obstacle detection, and vehicle tracking based on an encoder-decoder-based FCN, an extended Kalman filter, and camera, lidar, and radar sensor fusion for autonomous vehicles	Experimental results indicate that the proposed affordable, compact, and robust fusion system outperforms benchmark models and can be efficiently used in real-time for the vehicle’s environment perception
	[58]	CNN-based real-time semantic segmentation of 3D lidar data for autonomous vehicle perception based on the projection method and the adaptive break point detector method	Practical implementation and satisfactory speed and accuracy of the proposed method
	[59]	E2E self-driving algorithm using a CNN that predicts the vehicles’ longitudinal and lateral control values based on the input camera images and 2D lidar point cloud data	Experimental tests in the real-world complex urban environments show promising results
	[60]	Pedestrian recognition and tracking for autonomous vehicles using an SVM classifier and Velodyne 64 lidar data, generating alarms when pedestrians are detected on the road or close to curbs	The validity of the method was confirmed on the autonomous vehicle platform in two scenarios: when the vehicle is stationary and while driving

**Table 2:** Review of scientific papers on the application and analysis of the lidar system in the maritime sector.

	Reference	Description of Application	Conclusion
**Autonomous navigation and object detection**	[76]	Lidar as a part of the sensor system (absolute positioning, visual, audio, and remote sensing sensors) combined with artificial intelligence (AI) techniques for situational awareness in autonomous vessels	Several drawbacks of the current lidar technology are detected for application on autonomous vessels, including limited laser power due to eye-safety issues, lower operational ranges, expensive optics, and unsuitability for the harsh working environment
	[77]	Ship berthing information extraction based on the 3D lidar data using principal component analysis	The effectiveness of the proposed method in dynamic target recognition and safe ship berthing is confirmed by experimental validation on the ro-ro ship berthing
	[78]	Berthing perception framework for maritime autonomous surface ships based on the estimation of the vessel’s berthing speed, angle, distance, and other parameters from the 3D shipborne lidar data	The proposed method allows accurate berthing in real-time, as confirmed by experiments
	[79]	Low-cost lidar-based ship berthing and docking system, with a novel method of fusing lidar and GNSS positioning data	The usefulness of the proposed system in safe ship berthing is proven experimentally during several berthing maneuvers and compared to the GNSS-based navigational aid system
	[80]	Computer-aided method for bollard segmentation and position estimation from the 3D lidar point cloud data for autonomous mooring based on the 3D feature matching and mixed feature-correspondence matching algorithms	The proposed approach is validated on experimental mooring scenes with a robotic arm equipped with lidar
	[81]	Use of the dual-channel lidar for rotorcraft searching, positioning, tracking, and landing on a ship at sea based on the estimation of the azimuth angle, the distance of the ship relative to the rotorcraft, and the ship’s course	The simulation and experimental tests confirm the effectiveness of the developed method and associated models
	[82]	Algorithm for detecting objects on seas and oceans using lidar data for application on maritime vessels in different environmental conditions	A proven accurate object detection method called DBSCAN is used to cluster the data points
	[83]	Detection, monitoring, and classification of objects on seas and oceans based on the SVM classifier and the fusion of lidar and camera data	The proposed method is proven to be highly effective, with an overall accuracy of 98.7% for six classes
	[84]	Detection, classification, and mapping of objects on seas and oceans using an unmanned surface vehicle with four multi-beam lidar sensors and polygon representation methods	The ability to create a map of the environment with detected objects that are not in motion, with polygons being accurate to 20 cm using a 10 cm occupancy grid
**Monitoring ocean ecosystems**	[85]	A review of the development of profiling oceanographic lidars	The possibility of sea and ocean analysis and monitoring of animal species using lidar is described where these lidars can provide quantitative profiles of the optical properties of the water column to depths of 20–30 m in coastal waters and 100 m for a blue lidar in the open ocean
	[86]	Application of lidar for monitoring and mapping the marine coral reef ecosystems	Successful monitoring of fish, plankton, and coral reef distribution using 3D lidar data
	[87]	Spaceborne lidar for ocean observations	The usefulness of satellite lidar for observations of ocean ecosystems, particularly in combination with ocean color observations
**Mapping coastal areas**	[88]	A review of lidar application in creating shoreline and bathymetric maps	Lidar, combined with Global Positioning System (GPS), provides accurate topographical and bathymetric coastal maps, with 10–15 cm vertical accuracy, where best water penetration is achieved by using a blue-green laser with a wavelength of 530 nm
	[89]	Classification of large bodies of water using airborne laser scanning (ALS)	Automatic and efficient classification of water surfaces with an SVM classifier, with an accuracy of over 95% for most cases of coastal areas
	[90]	Mapping coastal terrains using unmanned aerial vehicle (UAV) lidar	High resolution and quality of topographic data (5–10 cm accuracy) of UAV lidar that outperforms UAV imagery in terms of ground coverage, point density, and the ability to penetrate through the vegetation
	[91]	Semi-automatic coastal waste detection and recognition using 3D lidar data	Possible classification of waste into plastic, paper, fabric, and metal
**Other** **applications**	[92]	Monitoring the dynamics of the upper part of the ocean by ship-lidar with the analysis of motion impact on lidar measurements	Measurement of waves, turbulence, and the impact of wind farms on the seas
	[93]	Doppler lidar-based data collection for offshore wind farms	High-resolution measuring of wind speed and direction at various altitudes for proper realization of offshore wind farms

## Data Availability

Not applicable.

## Abbreviations

2D	two-dimensional
3D	three-dimensional
ADAS	Advanced Driver Assistance System
AI	artificial intelligence
ALS	airborne laser scanning
AMCW	amplitude modulated continuous wave
APD	avalanche photodiode
BIM	building information modeling
BP-ANN	backpropagation artificial neural network
CMOS	complementary metal-oxide semiconductor
CNN	convolutional neural network
DAC	digital-to-analog converter
DBSCAN	density-based spatial clustering of applications with noise
DM-ISDNN	dual-modal instance segmentation deep neural network
E2E	end-to-end
FCN	fully convolutional neural network
FMCW	frequency modulated continuous wave
FOV	field of view
FRPDM	free-resolution probability distributions map
GIS	geographic information system
GMM	Gaussian mixture model
GNSS	Global Navigation Satellite System
GPS	Global Positioning System
GPU	graphics processing unit
HMM	hidden Markov model
IDW	inverse distance weighted
IMM	interacting multiple model
IMU	inertial measurement unit
KITTI	Karlsruhe Institute of Technology and Toyota Technological Institute
lidar	light detection and ranging
LoDNN	Lidar-only Deep Neural Network
MEMS	micro-electromechanical system
MLS	mobile laser scanning
MPE	maximum permissible exposure
MSTV	multiscale tensor voting
OPA	optical phased array
PMT	photomultiplier tube
radar	radio detection and ranging
RGB	red-green-blue
SiPM	silicon photomultiplier
SLAM	simultaneous localization and mapping
SNR	signal-to-noise ratio
SPAD	single-photon avalanche photodiode
SVM	support vector machine
TDC	time-to-digital converter
TLS	terrestrial laser scanning
ToF	time of flight
UAV	unmanned aerial vehicle
WND	weighted neighboring difference
WSMLR	weakly supervised metric learning

## References

[1] Li Y., Ibanez-Guzman J. (2020). Lidar for Autonomous Driving: The Principles, Challenges, and Trends for Automotive Lidar and Perception Systems. IEEE Signal Process. Mag..

[2] Frost & Sullivan (2016). LiDAR: Driving the Future of Autonomous Navigation.

[3] Fernandez Diaz J.C., Carter W.E., Shrestha R.L., Glennie C.L., Pelton J.N., Madry S., Camacho-Lara S. (2013). Lidar Remote Sensing. Handbook of Satellite Applications.

[4] Royo S., Ballesta-Garcia M. (2019). An Overview of Lidar Imaging Systems for Autonomous Vehicles. Appl. Sci..

[5] Yoo H.W., Druml N., Brunner D., Schwarzl C., Thurner T., Hennecke M., Schitter G. (2018). MEMS-Based Lidar for Autonomous Driving. Elektrotechnik Inf..

[6] Stann B.L., Dammann J.F., Giza M.M. (2016). Progress on MEMS-Scanned Ladar. Proceedings of the Laser Radar Technology and Applications XXI, Baltimore, MD, USA, 19–20 April 2016.

[7] Khader M., Cherian S. (2020). An Introduction to Automotive LIDAR.

[8] McManamon P. (2015). Field Guide to Lidar.

[9] Behroozpour B., Sandborn P.A.M., Wu M.C., Boser B.E. (2017). Lidar System Architectures and Circuits. IEEE Commun. Mag..

[10] Jha A., Azcona F.J., Royo S. (2016). Frequency-Modulated Optical Feedback Interferometry for Nanometric Scale Vibrometry. IEEE Photonics Technol. Lett..

[11] Velodyne LiDAR (2019). Velodyne LiDAR Puck.

[12] Velodyne LiDAR (2021). Velodyne LiDAR Alpha Prime.

[13] RIEGL Laser Measurement Systems GmbH (2021). RIEGL VUX-1HA.

[14] Leica Geosystems (2017). Leica ScanStation P50.

[15] Brnelić A. (2021). Application of Laser Systems for Object Detection in the Modern Transportation Sector. Master’s Thesis.

[16] Velodyne LiDAR (2019). VLP-16 User Manual.

[17] Wang Y., Chen Q., Zhu Q., Liu L., Li C., Zheng D. (2019). A Survey of Mobile Laser Scanning Applications and Key Techniques over Urban Areas. Remote Sens..

[18] Williams K., Olsen M.J., Roe G.V., Glennie C. (2013). Synthesis of Transportation Applications of Mobile LIDAR. Remote Sens..

[19] Vosselman G., Maas H.-G. (2010). Airborne and Terrestrial Laser Scanning.

[20] Guan H., Li J., Cao S., Yu Y. (2016). Use of Mobile LiDAR in Road Information Inventory: A Review. Int. J. Image Data Fusion.

[21] Hecht J. (2018). Lidar for Self-Driving Cars. Opt. Photonics News.

[22] Gargoum S., El-Basyouny K. Automated Extraction of Road Features Using LiDAR Data: A Review of LiDAR Applications in Transportation. Proceedings of the 2017 4th International Conference on Transportation Information and Safety (ICTIS).

[23] Zou Q., Sun Q., Chen L., Nie B., Li Q. (2021). A Comparative Analysis of LiDAR SLAM-Based Indoor Navigation for Autonomous Vehicles. IEEE Trans. Intell. Transp. Syst..

[24] Heinzler R., Schindler P., Seekircher J., Ritter W., Stork W. Weather Influence and Classification with Automotive Lidar Sensors. Proceedings of the 2019 IEEE Intelligent Vehicles Symposium (IV).

[25] Peynot T., Underwood J., Scheding S. Towards Reliable Perception for Unmanned Ground Vehicles in Challenging Conditions. Proceedings of the 2009 IEEE/RSJ International Conference on Intelligent Robots and Systems.

[26] Miclea R.-C., Dughir C., Alexa F., Sandru F., Silea I. (2020). Laser and LIDAR in a System for Visibility Distance Estimation in Fog Conditions. Sensors.

[27] Li Y., Duthon P., Colomb M., Ibanez-Guzman J. (2021). What Happens for a ToF LiDAR in Fog?. IEEE Trans. Intell. Transp. Syst..

[28] Lin S.-L., Wu B.-H. (2021). Application of Kalman Filter to Improve 3D LiDAR Signals of Autonomous Vehicles in Adverse Weather. Appl. Sci..

[29] Wojtanowski J., Zygmunt M., Kaszczuk M., Mierczyk Z., Muzal M. (2014). Comparison of 905 Nm and 1550 Nm Semiconductor Laser Rangefinders’ Performance Deterioration Due to Adverse Environmental Conditions. Opto-Electron. Rev..

[30] Caltagirone L., Scheidegger S., Svensson L., Wahde M. Fast LIDAR-Based Road Detection Using Fully Convolutional Neural Networks. Proceedings of the 2017 IEEE Intelligent Vehicles Symposium (IV).

[31] Wu J., Xu H., Zhao J. (2020). Automatic Lane Identification Using the Roadside LiDAR Sensors. IEEE Intell. Transp. Syst. Mag..

[32] Caltagirone L., Bellone M., Svensson L., Wahde M. (2019). LIDAR–Camera Fusion for Road Detection Using Fully Convolutional Neural Networks. Robot. Auton. Syst..

[33] Barazzetti L., Previtali M., Scaioni M. (2020). Roads Detection and Parametrization in Integrated BIM-GIS Using LiDAR. Infrastructures.

[34] Chen L., Yang J., Kong H. Lidar-Histogram for Fast Road and Obstacle Detection. Proceedings of the 2017 IEEE International Conference on Robotics and Automation (ICRA).

[35] Zhang Y., Wang J., Wang X., Dolan J.M. (2018). Road-Segmentation-Based Curb Detection Method for Self-Driving via a 3D-LiDAR Sensor. IEEE Trans. Intell. Transp. Syst..

[36] Kumar P., McElhinney C.P., Lewis P., McCarthy T. (2013). An Automated Algorithm for Extracting Road Edges from Terrestrial Mobile LiDAR Data. ISPRS J. Photogramm. Remote Sens..

[37] Wolcott R.W., Eustice R.M. Visual Localization within LIDAR Maps for Automated Urban Driving. Proceedings of the 2014 IEEE/RSJ International Conference on Intelligent Robots and Systems.

[38] de Miguel M.Á., García F., Armingol J.M. (2020). Improved LiDAR Probabilistic Localization for Autonomous Vehicles Using GNSS. Sensors.

[39] Ilci V., Toth C. (2020). High Definition 3D Map Creation Using GNSS/IMU/LiDAR Sensor Integration to Support Autonomous Vehicle Navigation. Sensors.

[40] Hata A.Y., Wolf D.F. (2016). Feature Detection for Vehicle Localization in Urban Environments Using a Multilayer LIDAR. IEEE Trans. Intell. Transp. Syst..

[41] Kim K.-W., Jee G.-I. (2020). Free-Resolution Probability Distributions Map-Based Precise Vehicle Localization in Urban Areas. Sensors.

[42] Wang H., Wang Z., Lin L., Xu F., Yu J., Liang H. (2021). Optimal Vehicle Pose Estimation Network Based on Time Series and Spatial Tightness with 3D LiDARs. Remote Sens..

[43] Xue H., Fu H., Dai B. (2019). IMU-Aided High-Frequency Lidar Odometry for Autonomous Driving. Appl. Sci..

[44] Yang B., Fang L., Li Q., Li J. (2012). Automated Extraction of Road Markings from Mobile Lidar Point Clouds. Photogramm. Eng. Remote Sens..

[45] Guan H., Li J., Yu Y., Ji Z., Wang C. (2015). Using Mobile LiDAR Data for Rapidly Updating Road Markings. IEEE Trans. Intell. Transp. Syst..

[46] Landa J., Prochazka D. (2014). Automatic Road Inventory Using LiDAR. Procedia Econ. Financ..

[47] Tan M., Wang B., Wu Z., Wang J., Pan G. (2016). Weakly Supervised Metric Learning for Traffic Sign Recognition in a LIDAR-Equipped Vehicle. IEEE Trans. Intell. Transp. Syst..

[48] Gargoum S., El-Basyouny K., Sabbagh J., Froese K. (2017). Automated Highway Sign Extraction Using Lidar Data. Transp. Res. Rec..

[49] Zhao J., Xu H., Liu H., Wu J., Zheng Y., Wu D. (2019). Detection and Tracking of Pedestrians and Vehicles Using Roadside LiDAR Sensors. Transp. Res. Part C Emerg. Technol..

[50] Wu J. (2018). An Automatic Procedure for Vehicle Tracking with a Roadside LiDAR Sensor. ITE J..

[51] Li B., Zhang T., Xia T. Vehicle Detection from 3D Lidar Using Fully Convolutional Network. Proceedings of the Robotics: Science and Systems XII.

[52] Asvadi A., Garrote L., Premebida C., Peixoto P., Nunes J.U. (2018). Multimodal Vehicle Detection: Fusing 3D-LIDAR and Color Camera Data. Pattern Recognit. Lett..

[53] Schlosser J., Chow C.K., Kira Z. Fusing LIDAR and Images for Pedestrian Detection Using Convolutional Neural Networks. Proceedings of the 2016 IEEE International Conference on Robotics and Automation (ICRA).

[54] Gao H., Cheng B., Wang J., Li K., Zhao J., Li D. (2018). Object Classification Using CNN-Based Fusion of Vision and LIDAR in Autonomous Vehicle Environment. IEEE Trans. Ind. Inform..

[55] Nowak T., Ćwian K., Skrzypczyński P. (2021). Real-Time Detection of Non-Stationary Objects Using Intensity Data in Automotive LiDAR SLAM. Sensors.

[56] Geng K., Dong G., Yin G., Hu J. (2020). Deep Dual-Modal Traffic Objects Instance Segmentation Method Using Camera and LIDAR Data for Autonomous Driving. Remote Sens..

[57] Shahian Jahromi B., Tulabandhula T., Cetin S. (2019). Real-Time Hybrid Multi-Sensor Fusion Framework for Perception in Autonomous Vehicles. Sensors.

[58] Kang D., Wong A., Lee B., Kim J. (2021). Real-Time Semantic Segmentation of 3D Point Cloud for Autonomous Driving. Electronics.

[59] Park M., Kim H., Park S. (2021). A Convolutional Neural Network-Based End-to-End Self-Driving Using LiDAR and Camera Fusion: Analysis Perspectives in a Real-World Environment. Electronics.

[60] Wang H., Wang B., Liu B., Meng X., Yang G. (2017). Pedestrian Recognition and Tracking Using 3D LiDAR for Autonomous Vehicle. Robot. Auton. Syst..

[61] Thakur R. (2016). Scanning LIDAR in Advanced Driver Assistance Systems and Beyond: Building a Road Map for next-Generation LIDAR Technology. IEEE Consum. Electron. Mag..

[62] Yeong D.J., Velasco-Hernandez G., Barry J., Walsh J. (2021). Sensor and Sensor Fusion Technology in Autonomous Vehicles: A Review. Sensors.

[63] Filgueira A., González-Jorge H., Lagüela S., Díaz-Vilariño L., Arias P. (2017). Quantifying the Influence of Rain in LiDAR Performance. Measurement.

[64] Fritsch J., Kühnl T., Geiger A. A New Performance Measure and Evaluation Benchmark for Road Detection Algorithms. Proceedings of the 16th International IEEE Conference on Intelligent Transportation Systems (ITSC 2013).

[65] RIEGL Laser Measurement Systems GmbH (2015). RIEGL VMX-450.

[66] Miyajima C., Takeda K. (2016). Driver-Behavior Modeling Using On-Road Driving Data: A New Application for Behavior Signal Processing. IEEE Signal Process. Mag..

[67] Behley J., Garbade M., Milioto A., Quenzel J., Behnke S., Stachniss C., Gall J. SemanticKITTI: A Dataset for Semantic Scene Understanding of LiDAR Sequences. Proceedings of the 2019 IEEE/CVF International Conference on Computer Vision (ICCV).

[68] Milioto A., Vizzo I., Behley J., Stachniss C. RangeNet ++: Fast and Accurate LiDAR Semantic Segmentation. Proceedings of the 2019 IEEE/RSJ International Conference on Intelligent Robots and Systems (IROS).

[69] Ester M., Kriegel H.-P., Sander J., Xu X. A Density-Based Algorithm for Discovering Clusters in Large Spatial Databases with Noise. Proceedings of the Second International Conference on Knowledge Discovery and Data Mining.

[70] Cai Z., Vasconcelos N. (2021). Cascade R-CNN: High Quality Object Detection and Instance Segmentation. IEEE Trans. Pattern Anal. Mach. Intell..

[71] Alzubaidi L., Zhang J., Humaidi A.J., Al-Dujaili A., Duan Y., Al-Shamma O., Santamaría J., Fadhel M.A., Al-Amidie M., Farhan L. (2021). Review of Deep Learning: Concepts, CNN Architectures, Challenges, Applications, Future Directions. J. Big Data.

[72] Lopac N., Hržić F., Vuksanović I.P., Lerga J. (2022). Detection of Non-Stationary GW Signals in High Noise from Cohen’s Class of Time–Frequency Representations Using Deep Learning. IEEE Access.

[73] Zhou X., Li Y., Liang W. (2021). CNN-RNN Based Intelligent Recommendation for Online Medical Pre-Diagnosis Support. IEEE/ACM Trans. Comput. Biol. Bioinform..

[74] Alhichri H., Alswayed A.S., Bazi Y., Ammour N., Alajlan N.A. (2021). Classification of Remote Sensing Images Using EfficientNet-B3 CNN Model with Attention. IEEE Access.

[75] Liu H., Jurdana I., Lopac N., Wakabayashi N. (2022). BlueNavi: A Microservices Architecture-Styled Platform Providing Maritime Information. Sustainability.

[76] Thombre S., Zhao Z., Ramm-Schmidt H., Vallet García J.M., Malkamäki T., Nikolskiy S., Hammarberg T., Nuortie H., Bhuiyan H.M.Z., Särkkä S. (2022). Sensors and AI Techniques for Situational Awareness in Autonomous Ships: A Review. IEEE Trans. Intell. Transp. Syst..

[77] Chen C., Li Y. (2021). Ship Berthing Information Extraction System Using Three-Dimensional Light Detection and Ranging Data. J. Mar. Sci. Eng..

[78] Hu B., Liu X., Jing Q., Lyu H., Yin Y. (2022). Estimation of Berthing State of Maritime Autonomous Surface Ships Based on 3D LiDAR. Ocean. Eng..

[79] Perkovič M., Gucma L., Bilewski M., Muczynski B., Dimc F., Luin B., Vidmar P., Lorenčič V., Batista M. (2020). Laser-Based Aid Systems for Berthing and Docking. J. Mar. Sci. Eng..

[80] Jindal M., Jha A., Cenkeramaddi L.R. (2022). Bollard Segmentation and Position Estimation from Lidar Point Cloud for Autonomous Mooring. IEEE Trans. Geosci. Remote Sens..

[81] Zeng T., Wang H., Sun X., Li H., Lu Z., Tong F., Cheng H., Zheng C., Zhang M. (2022). Dual-Channel LIDAR Searching, Positioning, Tracking and Landing System for Rotorcraft from Ships at Sea. J. Navig..

[82] Wessman M. (2018). Object Detection Using LIDAR in Maritime Scenarios. Master’s Thesis.

[83] Thompson D.J. (2017). Maritime Object Detection, Tracking, and Classification Using Lidar and Vision-Based Sensor Fusion. Master’s Thesis.

[84] Thompson D., Coyle E., Brown J. (2019). Efficient LiDAR-Based Object Segmentation and Mapping for Maritime Environments. IEEE J. Ocean. Eng..

[85] Churnside J.H. (2013). Review of Profiling Oceanographic Lidar. Optical Engineering.

[86] Pittman S.J., Costa B., Wedding L.M., Goodman J.A., Purkis S.J., Phinn S.R. (2013). LiDAR Applications. Coral Reef Remote Sensing: A Guide for Mapping, Monitoring and Management.

[87] Hostetler C.A., Behrenfeld M.J., Hu Y., Hair J.W., Schulien J.A. (2018). Spaceborne Lidar in the Study of Marine Systems. Annu. Rev. Mar. Sci..

[88] Klemas V. (2011). Beach Profiling and LIDAR Bathymetry: An Overview with Case Studies. J. Coast. Res..

[89] Smeeckaert J., Mallet C., David N., Chehata N., Ferraz A. (2013). Large-Scale Classification of Water Areas Using Airborne Topographic Lidar Data. Remote Sens. Environ..

[90] Lin Y.-C., Cheng Y.-T., Zhou T., Ravi R., Hasheminasab S.M., Flatt J.E., Troy C., Habib A. (2019). Evaluation of UAV LiDAR for Mapping Coastal Environments. Remote Sens..

[91] Ge Z., Shi H., Mei X., Dai Z., Li D. (2016). Semi-Automatic Recognition of Marine Debris on Beaches. Sci. Rep..

[92] Wolken-Möhlmann G., Gottschall J., Lange B. (2014). First Verification Test and Wake Measurement Results Using a SHIP-LIDAR System. Energy Procedia.

[93] Pichugina Y.L., Banta R.M., Brewer W.A., Sandberg S.P., Hardesty R.M. (2012). Doppler Lidar–Based Wind-Profile Measurement System for Offshore Wind-Energy and Other Marine Boundary Layer Applications. J. Appl. Meteorol. Climatol..

[94] Jurdana I., Lopac N., Wakabayashi N., Liu H. (2021). Shipboard Data Compression Method for Sustainable Real-Time Maritime Communication in Remote Voyage Monitoring of Autonomous Ships. Sustainability.

[95] Munim Z.H. (2019). Autonomous Ships: A Review, Innovative Applications and Future Maritime Business Models. Supply Chain. Forum Int. J..

[96] Felski A., Zwolak K. (2020). The Ocean-Going Autonomous Ship—Challenges and Threats. J. Mar. Sci. Eng..

[97] Dujmović J., Krljan T., Lopac N., Žuškin S. (2022). Emphasis on Occupancy Rates in Carbon Emission Comparison for Maritime and Road Passenger Transportation Modes. J. Mar. Sci. Eng..

[98] Lee P.T.-W., Kwon O.K., Ruan X. (2019). Sustainability Challenges in Maritime Transport and Logistics Industry and Its Way Ahead. Sustainability.

[99] Lopac N., Jurdana I., Lerga J., Wakabayashi N. (2021). Particle-Swarm-Optimization-Enhanced Radial-Basis-Function-Kernel-Based Adaptive Filtering Applied to Maritime Data. J. Mar. Sci. Eng..

